# The Distribution and Physiological Effects of the Myoinhibiting Peptides in the Kissing Bug, *Rhodnius Prolixus*

**DOI:** 10.3389/fnins.2012.00098

**Published:** 2012-07-06

**Authors:** Angela B. Lange, Uzma Alim, Hans Peter Vandersmissen, Akira Mizoguchi, Jozef Vanden Broeck, Ian Orchard

**Affiliations:** ^1^Department of Biology, University of Toronto MississaugaMississauga, ON, Canada; ^2^Animal Physiology and Neurobiology, Katholieke Universiteit LeuvenLeuven, Belgium; ^3^Division of Biological Science, Graduate School of Science, Nagoya UniversityNagoya, Japan

**Keywords:** insect, salivary gland, reproductive tissues, immunohistochemistry, muscle contraction, receptor assay

## Abstract

The myoinhibiting peptides (MIPs), also designated as allatostatin-Bs or prothoracicostatic peptides in some insects, are neuropeptides that are characterized by two tryptophan (W) residues at the C-terminal, denoted as the W(X_6_)Wamide motif. They are believed to be the ancestral ligands for the *Drosophila* sex peptide (SP) receptor. Physiological functions of MIPs include the inhibition of contraction of insect visceral muscles, in addition to allatostatic and prothoracicostatic activities. The MIP precursor in *Rhodnius prolixus* encodes MIPs that have an unusual W(X_7_)Wamide motif. In the present study, MIP-like immunoreactivity was detected within neurons in the central nervous system and within the innervation to the salivary glands, hindgut, and female and male reproductive systems of adult *R. prolixus*. The effects of peptides with the unusual W(X_7_)Wamide motif (Rhopr-MIP-4) and with the typical W(X_6_)Wamide motif (Rhopr-MIP-7) were tested for physiological activity on *R. prolixus* hindgut contractions. Both peptides reduce the frequency and amplitude of hindgut contractions in a dose-dependent manner. In addition, both peptides activate the *Drosophila* SP receptor. The MIP/SP receptors are therefore activated by peptides with the unusual W(X_7_)Wamide motif.

## Introduction

Various insect neuropeptide families are myoinhibitory when tested against contractions of visceral muscle, but might also have other biological functions (see Nässel and Winther, 2010). The first member of one such family was identified by Schoofs et al. (1991) and termed *Locusta* myoinhibiting peptide (Lom-MIP). This peptide (AWQDLNAGWamide) was found to inhibit spontaneous contractions of locust hindgut and oviduct, and processes immunoreactive for Lom-MIP were subsequently found to be associated with both of these tissues (Schoofs et al., 1991, 1996). Additional members of this family with the C-terminal consensus sequence W(X_6_)Wamide, and some resemblance to vertebrate galanin, have since been identified in a number of insect species, including *Manduca sexta*, *Bombyx mori*, *Periplaneta americana*, *Blattella germanica*, *Drosophila melanogaster*, *Gryllus bimaculatus*, *Carausius morosus*, and *Tribolium castaneum* (Blackburn et al., 1995, 2001; Lorenz et al., 1995, 2000; Hua et al., 1999; Predel et al., 2001; Williamson et al., 2001; Baggerman et al., 2002; Aguilar et al., 2006; Li et al., 2008), as well as in crustacean and molluscan species (Moroz et al., 2006; Fu et al., 2007). No members of this family have been reported in genome or peptidomic searches of *Apis mellifera*, *Nasonia vitripennis*, or *Acromyrmex echinatior* or other leaf-cutting ant species (see Hauser et al., 2010; Nygaard et al., 2011). Some MIPs have been designated as allatostatin-Bs (AST-Bs) because they inhibit juvenile hormone production in the corpora allata of *G. bimaculatus* (Lorenz et al., 1995) or as prothoracicostatic peptides (PTSPs) because they suppress ecdysteroid genesis in the prothoracic glands of *B. mori* (Hua et al., 1999; Yamanaka et al., 2010). Centrally, MIPs appear to play a role in the abdominal circuits associated with ecdysis behavior in *M. sexta* and *D. melanogaster* (Davis et al., 2003; Kim et al., 2006a,b; Santos et al., 2007), and in *Leucophaea maderae* in multiple brain circuits, including the circadian system (Schulze et al., 2012).

Although originally characterized by the W(X_6_)Wamide motif, these MIPs/AST-Bs/PTSPs, hereafter referred to as MIPs, are now known to also include peptides with a W(X_7_)Wamide motif. Thus, the MIP transcripts in *Acyrthosiphon pisum*, *Rhodnius prolixus*, and *Daphnia pulex* code for both W(X_6_)Wamide and W(X_7_)Wamide peptides (Huybrechts et al., 2010; Dircksen et al., 2011; Ons et al., 2011). In *R. prolixus*, the prepropeptide contains 12 MIP copies, 9 of which are W(X_7_)Wamides (Ons et al., 2011). This is an interesting discovery since MIPs appear to be the ancestral ligand for the *Drosophila* sex peptide (SP) receptor (Kim et al., 2010; Poels et al., 2010; Yamanaka et al., 2010) and SP has a W(X_8_)W at its core. The two Trp residues of MIPs and SP are necessary for total activation of the receptor (Kim et al., 2010; Poels et al., 2010).

In light of the unusual W(X_7_)Wamide motif in multiple copies of Rhopr-MIPs we have verified the Rhopr-MIP transcript, used immunohistochemistry to examine the distribution of Rhopr-MIPs in the central nervous system (CNS) and peripheral nervous system of *R. prolixus*, and also tested the effects of selected Rhopr-MIPs [one W(X_6_)Wamide and one W(X_7_)Wamide] on contractions of hindgut. In addition, we have tested these Rhopr-MIPs on the *Drosophila* SP receptor using a heterologous receptor assay to verify whether both the W(X_6_)Wamide and W(X_7_)Wamide types of MIPs are capable of activating the same receptor. *Drosophila* SP receptor was chosen as a model in this assay since the Rhopr-MIP receptor is currently still elusive. It is currently the only MIP receptor type known in insects. We were also interested in gaining some insight into the co-evolution of receptor-ligands and into the degrees of freedom in the spacing of the key W residues for receptor activation.

## Materials and Methods

### Animals

*Rhodnius prolixus* used in this study were taken from a long-standing colony maintained at the University of Toronto Mississauga. These insects were fed on defibrinated rabbits’ blood and maintained under high humidity in incubators at 25°C. Immunohistochemistry and physiological assays were performed on tissues taken from female adult and male *R. prolixus* that were fed as fifth instars ∼5–7 weeks prior to the experiments. Also, animals that contained material within their hindgut were selected for the hindgut contraction assays.

### Chemicals

Rhopr-MIP-4(AWSDLQSSGWamide) and Rhopr-MIP-7(AWNSLHGGWamide) were custom synthesized by GenScript (Piscataway, NJ, USA) and reconstituted with double distilled water into a stock solution of 10^−3^ M. The stock solution was stored as 10 μL aliquots at −20°C until working dilutions were made using *R. prolixus* saline (150 mM NaCl, 8.6 mM KCl, 2 mM CaCl_2_, 8.5 mM MgCl_2_, 4 mM NaHCO_3_, 5 mM HEPES, 34 mM glucose; pH 7.0).

### Immunohistochemistry

Tissues were dissected under *R. prolixus* saline and fixed at 4°C paraformaldehyde in Millonig’s buffer pH 7.4 (0.13 M NaH_2_PO_4_·H_2_O, 0.1 M NaOH, 1.2% glucose, 0.3 mM CaCl_2_). The tissues were processed for immunohistochemistry as previously described (Kwok et al., 2005; Te Brugge et al., 2005) using a 1:1000 mouse anti-MIP primary antibody (#7B3) made up in PBS, containing 0.4% Triton X-100, 2% BSA, and 2% NGS. This antibody is one of the monoclonal antibodies produced in the previous study (Yamanaka et al., 2010) and shows similar affinity to *M. sexta* MIP I, II, IV, and V and only 10× less affinity for Rhopr-MIP-4 and Rhopr-MIP-7 (Mizoguchi, unpublished result). The secondary antibody was a Cy3-labeled goat anti-mouse immunoglobulin (Jackson ImmunoResearch Laboratories, West Grove, PA, USA) used at a dilution of 1:600 with 10% NGS in PBS on a shaker for 48 h at 4°C. Control experiments were performed in which the antiserum was preincubated for 18 h with either 10^−5^ M Rhopr-MIP-4 or Rhopr-MIP-7 prior to use. The preparations were mounted in 100% glycerol following dehydration, and were viewed with a Nikon Optiphot 2 Epifluorescence Microscope (Nikon Corporation, Tokyo, Japan). Pictures were taken with a Zeiss LSM 510 Confocal Laser Microscope (Carl Zeiss, Jena, Germany) and a Qimaging Monochrome QICAM 10-bit digital camera (Qimaging, Burnaby, Canada).

### *In silico R. prolixus* precursor analysis

Geneious Pro 3.85 Software^1^ and BLAST analysis^2^ was used to analyze the Rhopr-MIP precursor in the *R. prolixus* genomic database. Geneious Pro 3.85 Software was also used to determine the consensus sequence for the nine Rhopr-MIPs.

### *R. prolixus* hindgut contraction assay

Hindguts from female adult *R. prolixus* were dissected under *R. prolixus* saline, leaving a small portion of the ventral cuticle attached to the posterior end. The preparation was placed in a shallow trough molded into a Sylgard-coated petri dish. The preparation was secured to the dish by pinning the cuticle using minuten pins. The anterior end of the hindgut was attached to an AE875 miniature force transducer (Aksjeselskapet Mikro-Elektronikk, Norway) using a strand of silk. Muscle activity was monitored on a linear flat-bed chart recorder connected to a force transducer through an amplifier. Isolated hindguts were maintained in 200 μL of saline at room temperature. Working concentrations of Rhopr-MIP-4 or Rhopr-MIP-7 were applied by removing 100 μL of saline and replacing it with the same volume of peptide that yielded final concentrations of 10^−11^ to 10^−6^ M. The peptides were washed from the preparation using saline between trials. The effects of the peptides on hindgut contractions were monitored over a 5 min time period.

Responses to Rhopr-MIP-4 and Rhopr-MIP-7 were quantified by dividing the frequency of contractions during the second minute after application, by the frequency of contractions during the 1 min interval prior to peptide application.

### *Drosophila melanogaster* sex peptide receptor assay

Drome-SPR expression vector (described by Poels et al., 2010) transfected and mock transfected (negative control) CHO-WTA11 cells (expressing apoaequorin and G_α16_) were grown until 90% confluency, detached using PBS with 100 μM EDTA, spun (6 min, 150 g) and resuspended at 5 × 10^6^ cells/mL in D-MEM/F-12 without phenol red supplemented with 0.1% BSA. “Coelenterazine h” (Invitrogen) was added to a concentration of 5 μM and the cells were incubated in the dark at room temperature for 3–4 h. After coelenterazine loading, cells were diluted 10-fold and incubated for 30 min. Test compounds were dissolved in 50 μL D-MEM/F-12 supplemented with 0.1% BSA and dispensed in triplicate into the wells of a white 96-well plate. Light emission was recorded (*Mithras* LB940, Berthold) for 30 s immediately after injection of 50 μL cell suspension into each well. Cells were then lysed by a second injection of 50 μL 0.3% Triton X-100 and monitored during 8 s. Data were collected using MikroWin2000 (Mikrotek Laborsysteme Gmbh).

## Results

### Immunohistochemistry

Like all antibodies used in immunohistochemistry, the MIP-antibody could potentially cross-react with antigens other than the specific one it was generated against (including other members of the same family), and hence the term MIP-like immunoreactivity. However, the MIP-antibodies generated have been used to visualize MIPs across diverse insect species, and in these species the staining matches the expression of the MIP transcript (see Kim et al., 2010). Using ELISA we have found the #7B3 antibody to have similar affinity for both Rhopr-MIP-4 and Rhopr-MIP-7, although ∼10× less affinity than for Manse-MIP-1. Furthermore, pre-incubation of the anti-MIP primary antibody in this study with either 10^−5^ M Rhopr-MIP-4 or Rhopr-MIP-7 eliminated all immunoreactive staining suggesting that the observed staining reported below was a result of MIP-like peptide.

### Immunoreactivity in the central nervous system and associated neurohemal areas

**Figure 1:**
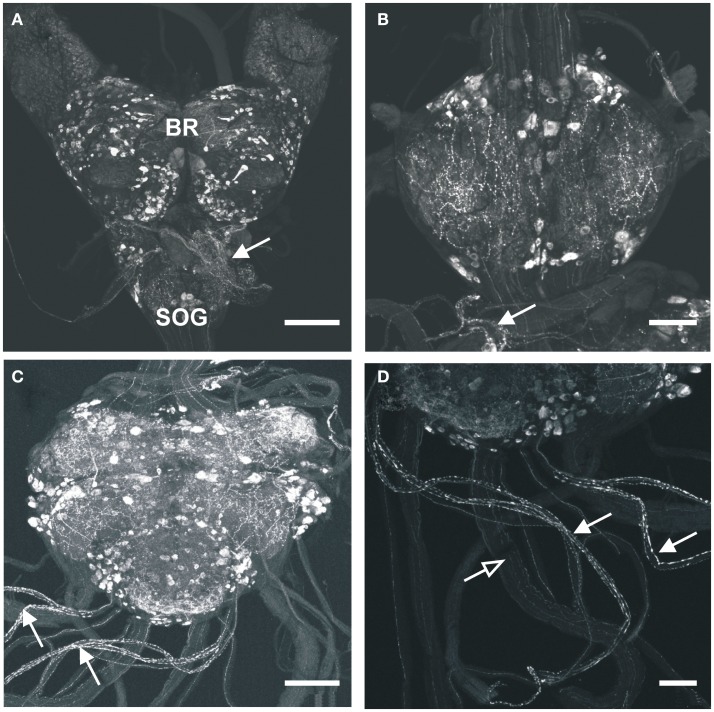
**Myoinhibiting peptides-like immunoreactivity is associated with cells and processes within the central nervous system of *R. prolixus***. **(A)** MIP-like immunoreactivity in cells and processes of the brain (BR) and sub-esophageal ganglion (SOG). Immunoreactive processes are also seen on the corpus cardiacum and corpus allatum (arrow). **(B)** MIP-like immunoreactive cells within the prothoracic ganglion as well as processes within the connectives. A prominent neurohemal area (arrow) is evident on the first thoracic nerve extending from the mesothoracic ganglion mass (MTGM). **(C,D)** MIP-like immunoreactivity in cells and processes within the MTGM with axons present in the abdominal nerves and trunk nerves. Note the neurohemal areas present on abdominal nerves 1 and 2 [**(C,D)**, closed arrows] and axons within the trunk nerves [**(D)**, open arrows]. Scale bars: **(A,C)**, 200 μm; **(B,D)**, 100 μm.

A large number of MIP-like immunoreactive neurons are distributed throughout all ganglia of the CNS of *R. prolixus*. These typically occur as bilaterally symmetrical cell bodies that produce brightly stained immunoreactive neuropil processes in many regions of the CNS (Figure 1). The number of MIP-like immunoreactive neurons was highest in the brain (several hundred) and mesothoracic ganglion mass (MTGM, ∼300), relative to the sub-esophageal ganglion (SOG) and prothoracic ganglion (PRO). Although a detailed analysis of the distribution of neurons within the CNS was not performed on these whole mounts (and our interest lies mainly in the periphery), it was clear that within the brain there were extensive fine, beaded neuropile processes associated with the optic lobe, antennal lobe, and medial lobes, with axons traversing the protocerebral bridge. Cell bodies were present throughout most of the brain regions, including the optic lobe/brain junction, the protocerebrum, deutocerebrum, and tritocerebrum. MIP-like immunoreactive axons were observed in the connectives between ganglia and in peripheral nerves, where they terminated in neurohemal areas (Figures 1B–D). Staining was observed in processes and varicosities of the corpus cardiacum (CC) which also extended along the anterior regions of the dorsal vessel (Figure 1A, arrow). An extensive neurohemal site displaying MIP-like immunoreactive staining was observed on branches of the first peripheral nerve leaving the mesothoracic neuromere (Figure 1B, arrow). MIP-like immunoreactive processes were present in each of the five abdominal nerves, resulting in particularly extensive neurohemal sites on abdominal nerves 1 and 2 (Figures 1C,D). Immunoreactive axons were also observed in the paired trunk nerves leaving the posterior MTGM (Figure 1D), with three strongly immunoreactive and five faintly immunoreactive processes present in each.

### MIP-like immunoreactivity associated with peripheral tissues

**Figure 2:**
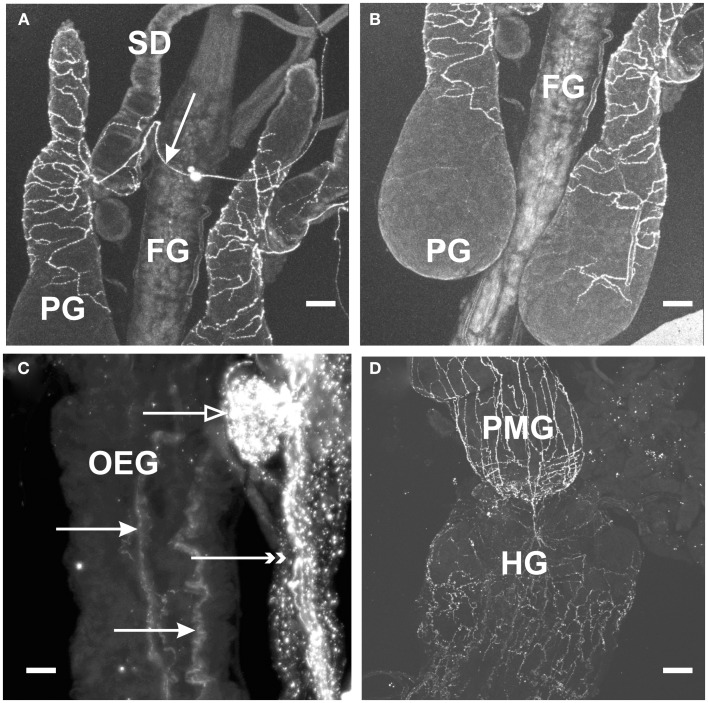
**Myoinhibiting peptides-like immunoreactivity is associated with peripheral tissue of *R. prolixus***. **(A,B)** MIP-like immunoreactive axons are present in the salivary gland nerve (arrow) with immunoreactive processes extending over the salivary gland duct (SD) and the principal gland (PG). No immunoreactive processes are seen on the foregut (FG). **(C)** MIP-like immunoreactive processes are seen on the esophagus (closed arrows) as well as processes on the dorsal vessel (double arrowheads) and covering the corpora allata (open arrow). **(D)** MIP-like immunoreactive processes extend from the hindgut (HG) onto the posterior midgut (PMG). Scale bars: **(A,B,D)**, 100 μm; **(C)**, 25 μm.

With regard to the digestive system, MIP-like immunoreactive processes were observed over the surface of the esophagus, posterior midgut, and anterior hindgut (Figures 2C,D), with processes petering out as they projected to the posterior hindgut. No immunoreactive processes were observed on the foregut, or the anterior midgut. MIP-like immunoreactivity was present within axons in the salivary gland nerve and immunoreactive processes were seen over the principal gland, accessory gland, and principal duct of this organ (Figures 2A,B).

**Figure 3:**
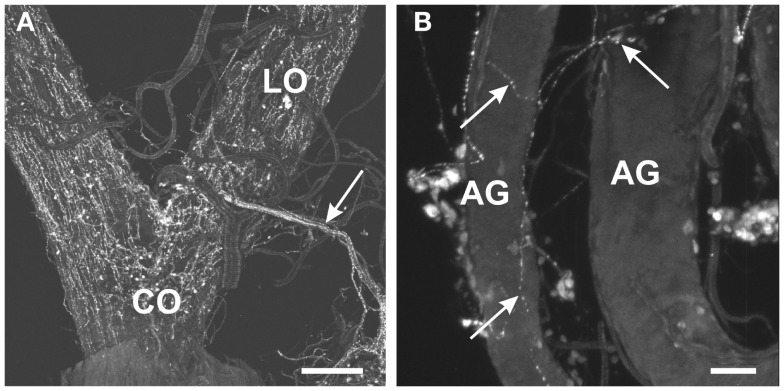
**Myoinhibiting peptides-like immunoreactivity is associated with the female and male reproductive tissues of *R. prolixus***. **(A)** MIP-like axons are present in the oviducal nerve (arrow) and immunoreactive processes are associated with both the lateral oviducts (LO) and common oviducts (CO). **(B)** A fine network of MIP-like immunoreactive processes (arrows) is evident around the male accessory glands (AG). Scale bars: **(A)**, 200 μm; **(B)**, 50 μm.

Within the female reproductive system, brightly stained MIP-like immunoreactive processes were present on the lateral and common oviduct (Figure 3A). Intensely stained varicosities were distributed over the surface of these structures. Also, three MIP-like immunoreactive axons were present in the branches from the trunk nerves which innervate the oviducts (Figure 1D, arrow in Figure 3A). There was no staining on the ovaries, spermathecae, cement gland, or bursa.

In the male reproductive tissue, a fine network of MIP-like immunoreactive processes were observed on the accessory glands, but not on other regions of the male reproductive system (Figure 3B, arrows).

In examining the dorsal vessel, heart, and alary muscles, MIP-like immunoreactivity was observed along the anterior dorsal vessel near the CC (Figures 1A and 2C), but was not present within the innervation to the posterior heart or alary muscles.

### *In silico* results

An *in silico* approach was used to verify the Rhopr-MIP precursor identified previously in *R. prolixus* by Ons et al. (2011; Table 1). Three modifications were determined. Firstly, one amino acid within the precursor and located in one of the predicted MIPs was determined to be a threonine (T) residue and not an arginine (R) residue (here labeled Rhopr-MIP-8). Secondly, an encoded peptide listed in the *de novo* sequenced table of Ons et al. (2011) is more likely to be EPAWQNLKGLWamide rather than the listed EPAWQNLKGLWGamide since the G is the amidation signal (here labeled Rhopr-MIP-9). Thirdly, one of the predicted peptides was missed from the table in Ons et al. (2011), namely, GWKDMQSSGWamide (here termed Rhopr-MIP-5).

**Table 1:** ***Rhodnius prolixus* MIPs**.

Rhopr-MIP	Designation	W(X_7_)W/W(X_6_)W
SWKDLQSSGWamide	Rhopr-MIP-1	W(X_7_)W
GWKDMQTVGWamide	Rhopr-MIP-2	W(X_7_)W
AWTDLPSSGWamide	Rhopr-MIP-3	W(X_7_)W
AWSDLQSSGWamide	Rhopr-MIP-4	W(X_7_)W
GWKDMQSSGWamide	Rhopr-MIP-5	W(X_7_)W
AWSDLQSSGWamide	Rhopr-MIP-4	W(X_7_)W
AWSDLQSSGWamide	Rhopr-MIP-4	W(X_7_)W
DWKDMQSSGWamide	Rhopr-MIP-6	W(X_7_)W
AWSDLQSSGWamide	Rhopr-MIP-4	W(X_7_)W
AWNSLHGGWamide	Rhopr-MIP-7	W(X_6_)W
TADWGSFTGSWamide	Rhopr-MIP-8	W(X_6_)W
EPAWQNLKGLWamide	Rhopr-MIP-9	W(X_6_)W

Table 1 depicts the 12 encoded peptides in the Rhopr-MIP precursor [3 of which are W(X_6_)Wamide and 9 of which are W(X_7_)Wamide] that are designated in this study as Rhopr-MIP-1 to Rhopr-MIP-9 (because of replicate copies). The consensus sequence found using Geneious Pro demonstrates the amino acids that are most conserved amongst the Rhopr-MIPs and indicates the consensus sequence to be most similar to Rhopr-MIP-4, with a W(X_7_)Wamide motif.

### Physiology

#### Effects of Rhopr-MIP-4 and Rhopr-MIP-7 on hindgut muscle contractions

**Figure 4:**
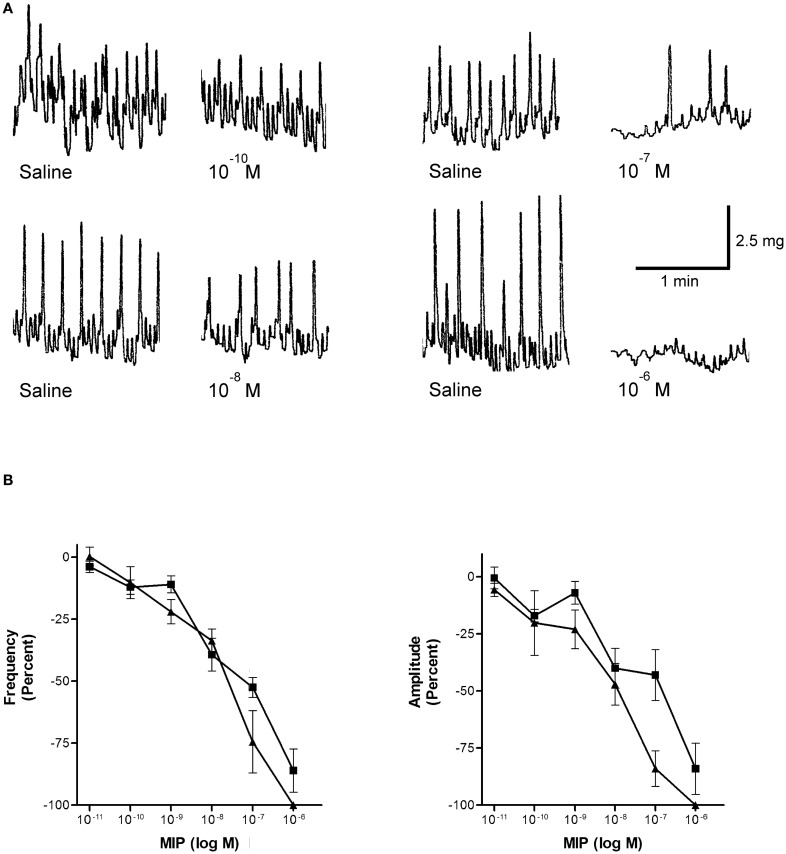
**Rhopr-MIPs inhibit the frequency and amplitude of hindgut contraction**. **(A)** Sample traces of Rhopr-MIP-7 [W(X_6_)Wamide] on spontaneous contractions of *R*. prolixus hindgut contractions. The traces show the saline control followed by the addition of Rhopr-MIP-7 1 min after the addition. **(B)** Rhopr-MIP-4 [W(X_7_)Wamide; triangles] and Rhopr-MIP-7 [W(X_6_)Wamide; squares] inhibit the frequency and amplitude of spontaneous hindgut contractions of *R. prolixus* in a dose-dependent manner. Symbols are mean ± SE of five to six preparations.

*Rhodnius prolixus* hindgut contractions are composed of spontaneous longitudinal and circular muscle contractions of variable strength and frequency. Rhopr-MIP-4 decreases the amplitude and frequency of spontaneous hindgut contractions in a dose-dependent manner, with a threshold concentration of about 0.1 nM, and an IC_50_ value of ∼10 nM for amplitude and 20 nM for frequency (Figure 4). Rhopr-MIP-4 was also capable of completely inhibiting contractions at 1 μM. The effects of Rhopr-MIP-4 were reversible, and spontaneous contractions resumed a few minutes after washing with saline. Rhopr-MIP-7 also decreases spontaneous hindgut contractions in a reversible and dose-dependent manner with a threshold concentration of 0.1 nM, and an IC_50_ of ∼100 nM for amplitude and 20 nM for frequency (Figure 4). Spontaneous contractions were not completely inhibited at 1 μM.

#### Effects of Rhopr-MIPs on the *Drosophila* sex peptide receptor

**Figure 5:**
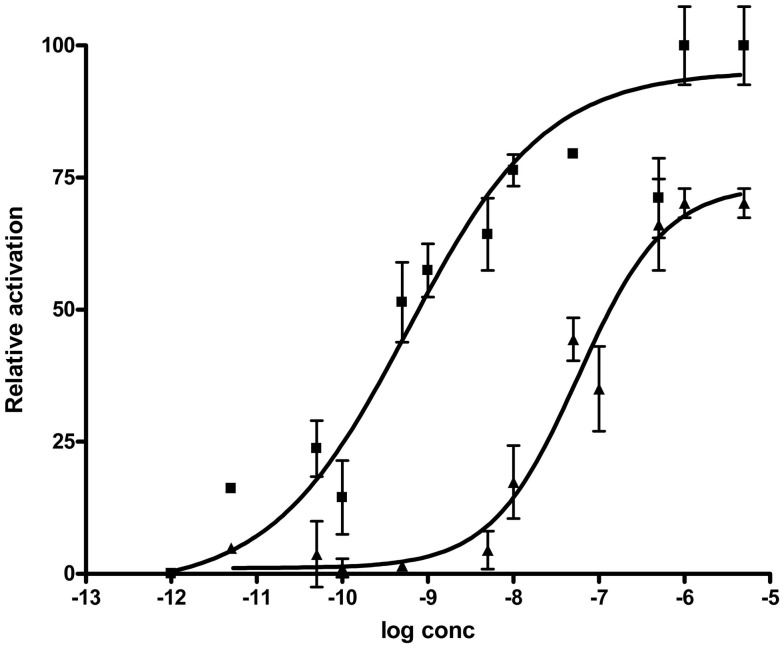
***Drosophila* SPR is activated by both Rhopr-MIP-7 [W(X_6_)Wamide; squares] and Rhopr-MIP-4 [W(X_7_)Wamide; triangles]**. CHO-cells coexpressing aequorin, G_α16_, and *Drosophila* SPR show a dose-dependent increase of their intracellular Ca^2+^-concentration (detected as aequorin luminescence) when challenged with Rhopr-MIP-4 or Rhopr-MIP-7. Data points represent mean ± SEM of three independent measurements done in triplicate and are given in percentage of the maximal response. The zero response level corresponds to treatment with buffer only.

The activity profiles of Rhopr-MIP-4 and -7 were tested in the functional aequorin-based Drome*-*SPR assays (Figure 5). Both of the selected Rhopr-MIPs are potent agonists for the Drome-SPR, with a subnanomolar EC_50_ for Rhopr-MIP-7 (0.59 nM) and a 100-fold higher EC_50_ for Rhopr-MIP-4 (53 nM). In the same assay, Drome-SP had an EC_50_ of about 15 nM (Poels et al., 2010).

## Discussion

Myoinhibiting peptides-like immunoreactivity is present in a vast number of cell bodies (>1,000) and processes in the brain, SOG, PRO, and MTGM of adult *R. prolixus*. In comparison with *L. migratoria*, *M. sexta*, or *B. mori*, the CNS of *R. prolixus* has a much larger number of MIP-like immunoreactive neurons, indicating that MIPs might play important central roles as neurotransmitters/neuromodulators in *R. prolixus*. Such has also been proposed for the cockroach *L. maderae*, where ∼2,000 cells have been described (Schulze et al., 2012). Indeed, in this cockroach, there is a very wide distribution of MIP-like immunoreactive neurons in the brain, including co-localization with circadian pacemaker cells containing pigment-dispersing factor, leading the authors to suggest that MIPs may be involved in the circadian system, in the processing of chemosensory information and also in the coordination of feeding and locomotory centers (Schulze et al., 2012). Similarly, in *R. prolixus* one might speculate that the wide distribution of MIP-like immunoreactive neurons in the various regions of the brain suggests an involvement of MIPs in at least visual processing, and also the olfactory system. In *D. melanogaster*, MIPs (and many other peptides) are present in the antennal lobe, again suggesting an involvement in sensory processing (Carlsson et al., 2010), and in the lamina of the visual system, indicating an involvement with the circadian clock (Kolodziejczyk and Nässel, 2011a,b), and MIPs have also been shown to be involved in activating the central ecdysis motor program in *M. sexta* and *B. mori* (Davis et al., 2003; Kim et al., 2006a,b). The involvement of MIPs in the visual system and putative clock neurons has also been compared between *Calliphora vomitoria* and *D. melanogaster* (Kolodziejczyk and Nässel, 2011a).

In *R. prolixus*, MIP-like immunoreactive processes leave the CNS along many peripheral nerves, and also terminate in neurohemal sites on the CC, anterior part of the dorsal vessel and thoracic and abdominal nerves, suggesting that MIPs are also neurohormones. This is also suggested for some other insects. For example, the glandular lobe of the CC of *L. migratoria* also contains MIP-like immunoreactive processes, as does the CC of *L. maderae*, although none were observed in the corpora cardiaca/corpora allata (CC/CA) or neurohemal perisympathetic organs of *M. sexta* (Davis et al., 2003); however, *M. sexta* does have MIP-like immunoreactive epiprocteal glands on the proctodeal nerve, and the authors suggest that MIPs are released from these endocrine glands to inhibit the prothoracic glands during the molting cycle and facilitate the decline in ecdysteroid titer (Davis et al., 2003).

Myoinhibiting peptides-like immunoreactive processes are present in the salivary gland nerve, and in both of the trunk nerves leaving the posterior MTGM. These trunk nerves branch to become nerves projecting to the posterior abdominal segments, to the hindgut through the proctodeal nerve, and to reproductive organs (Te Brugge et al., 2005). The presence of MIP-like immunoreactivity in the salivary gland nerve and the trunk nerves is consistent with the presence of MIP-like immunoreactivity associated with the salivary glands, posterior midgut and hindgut, and reproductive organs.

As shown in *L. migratoria*, *M. sexta*, *B. germanica*, *and P. americana* (Schoofs et al., 1996; Predel et al., 2001; Davis et al., 2003; Aguilar et al., 2006), MIP-like immunoreactivity is detected in processes over the *R. prolixus* hindgut suggesting an involvement in intestinal physiological processes. In addition, MIP-like immunoreactivity is observed over the salivary glands in *R. prolixus*. MIPs have previously been identified within the innervation of the salivary glands of *L. migratoria* and the black-legged tick, *Ixodes scapularis* (Schoofs et al., 1996; Šimo et al., 2009). The *R. prolixus* salivary glands facilitate feeding by secreting saliva that counteracts components of the host’s hemostasis (Orchard and Te Brugge, 2002). Contractions of muscles surrounding the salivary glands may contribute to mixing salivary gland contents, as well as with secreting saliva from the principal gland (Orchard, 2006). The presence of MIP-like immunoreactivity in the salivary glands suggests that MIPs may have a potential role in modulating these activities of the salivary glands.

Myoinhibiting peptides-like immunoreactive axons are present in the nerves that projects to the oviducts, as well as in processes that extend over the lateral oviducts and the common oviduct. Similarly, Lom-MIP-like immunoreactive processes were observed in nerves innervating the *L. migratoria* oviduct (Schoofs et al., 1996). Thus MIPs may have a potential involvement in reproductive success. Contractions of the oviduct may be important during oviposition in order to propel eggs down into the bursa (Lange, 1990, 2009). The presence of MIP-like immunoreactivity within the *R. prolixus* male reproductive system, specifically in the accessory glands, demonstrates that MIPs may play a role in *R. prolixus* male reproductive processes. Insect male accessory glands guarantee the production of the spermatophore and seminal fluid, which enables the successful transfer of semen to the female. The duct of the accessory gland along with the vas deferens opens into the ampullary part of the ejaculatory duct (Chen, 1984). Also, during copulation, sperm which are stored in the seminal vesicle enter the ejaculatory duct where they are mixed with the glandular secretion and then transferred to the female genital canal (Chen, 1984). Therefore, MIPs may be involved in the regulation of secretion and transport of secretory contents to the ejaculatory duct in *R. prolixus*. Interestingly, no MIP-like immunoreactivity was found associated with male reproductive organs of *D. melanogaster*, *D. mojavensis*, *B. mori*, or *T. castaneum* (Kim et al., 2010).

As shown in many insects (and as indicated by their name) MIPs are inhibitory of visceral muscle contraction. Thus, in *R. prolixus*, MIPs inhibit contractions of the hindgut in a dose-dependent manner. This is true for Rhopr-MIP-4 and for Rhopr-MIP-7. This is an interesting observation, since Rhopr-MIP-4 possesses an unusual W(X_7_)Wamide motif, but still retains inhibitory activity. The thresholds for these Rhopr-MIPs were approximately the same. These threshold concentrations are lower than the threshold concentrations determined for the suppression of hindgut muscle contractions in *L. migratoria* (1.8 nM Lom-MIP), *M. sexta* (10 nM Mas-MIP III-VI), and *P. americana* (100 nM Pea-MIP; Schoofs et al., 1991; Blackburn et al., 2001; Predel et al., 2001). In addition, although Rhopr-MIP-4 has similar thresholds to Rhopr-MIP-7, Rhopr-MIP-4 appears to be more active with regard to IC_50_ and its ability to completely abolish contractions at 1 μM. The presence of MIP-like immunoreactivity in the *R. prolixus* posterior midgut and hindgut, and the inhibitory effects of Rhopr-MIP-4 and Rhopr-MIP-7 on hindgut contractions suggest that MIPs have a function in regulating various digestive processes such as the processing and movement of blood meals. The hindgut of *R. prolixus* has an important role during rapid post-feeding diuresis because hindgut contractions are required to expel hindgut contents (Te Brugge et al., 2008).

Interestingly, both Rhopr-MIPs are capable of activating the Drome*-*SPR. SP consists of 36 amino acids with a core W(X_8_)W but a different C-terminal to MIPs. Poels et al. (2010) and Kim et al. (2010) demonstrated that the two W residues of MIPs and SP are necessary for total receptor activation, and so SP and MIPs share similar structural requirements for SPR activation. Whilst the two families of peptides are structurally quite different except for the two W residues, SP contains a C-terminal disulfide bridge that is required for receptor activation and that might conserve the position of the two W residues at the secondary structure level. In addition, modeling predicts that both peptide families have a β-turn conformation that might be stabilized by the two W residues (Kim et al., 2010). Thus, MIPs are believed to be the ancestral ligands of the SPR in *Drosophila* (Kim et al., 2010; Poels et al., 2010; Yamanaka et al., 2010). The SPR is considered quite promiscuous, and we now show that it is also activated by MIPs with a W(X_7_)Wamide motif. The EC_50_ on the SPR is 100-fold lower, though, for the Rhopr-MIP-7 which has the W(X_6_)Wamide motif. Since there are no MIPs in *Drosophila* with the W(X_7_)Wamide motif, the activation of the SPR by Rhopr-MIP-4 is an indication that the receptor allows for some degree of freedom in the spacing of the key W residues. Taking into account the possibility of receptor-ligand co-evolution in *D. melanogaster*, it appears logical that the W(X_6_)Wamide-containing peptide, Rhopr-MIP-7, displays a lower EC_50_ for Drome-SPR than the W(X_7_)Wamide MIP-variant, Rhopr-MIP-4. Interestingly, the latter peptide has an EC_50_ that is only slightly higher than the EC_50_ of Drome-SP, which contains a W(X_8_)W signature in addition to a disulfide bridge in its C-terminal part.

Although a cognate MIP receptor has not yet been identified in *R. prolixus*, one can hypothesize that there may have been co-evolution of the MIP and its receptor, such that the receptor has equal or higher sensitivity to the W(X_7_)Wamide motif, which is the dominant form of the peptide (9 of 12 copies on the precursor). This latter situation would be comparable to the situation for the human gonadotropin releasing hormone (GnRH) where there are two GnRHs but only one receptor, and this has a higher affinity for GnRH I than for GnRH II (Lu et al., 2007). Alternatively, it cannot be excluded that in *R. prolixus* (and possibly also in other species) multiple MIP receptors are present which may each have a higher affinity toward the W(X_7_)Wamide motif or the more common W(X_6_)Wamide motif. In the case of glycoprotein hormone receptors in vertebrates, three receptors have evolved from a common ancestor, each of which is highly specific in its ligand recognition. In these cases, regions determining the ligand specificity have been identified and have been correlated with differential motifs in the hormones (Caltabiano et al., 2008). The genome data available for *R. prolixus* have not yet revealed a clear SPR homolog. Likely, future releases of the genome data and their annotations will allow the identification of this receptor. A detailed characterization of MIP-SPR signaling in this organism will only be possible when the SPR (or possibly, multiple receptors) has been identified.

In conclusion, this study demonstrates the presence and distribution of MIP-like immunoreactivity throughout neurons in the CNS, in neurohemal sites, and in the innervation to the hindgut, posterior midgut, salivary glands, and female and male reproductive systems, of adult *R. prolixus*. Physiological assays demonstrate that the native *R. prolixus* MIPs, Rhopr-MIP-4, and Rhopr-MIP-7, inhibit the frequency of *R. prolixus* hindgut muscle contractions, despite Rhopr-MIP-4 having an unusual W(X_7_)Wamide motif. In addition, both MIPs are active on the *Drosophila* SPR.

## Conflict of Interest Statement

The authors declare that the research was conducted in the absence of any commercial or financial relationships that could be construed as a potential conflict of interest.

## Footnotes

^1^www.geneious.com

^2^http://blast.ncbi.nlm.nih.gov/Blast.cgi
